# Oral health practices and prevalence of dental plaque and gingivitis among Indian adults

**DOI:** 10.1002/cre2.15

**Published:** 2016-01-28

**Authors:** P.K. Sreenivasan, K.V.V. Prasad, S. B. Javali

**Affiliations:** ^1^ Colgate‐Palmolive Technology Center Piscataway New Jersey 08855 USA; ^2^ Department of Community Dentistry SDM College of Dental Sciences Dharwad India; ^3^ Associate Professor in Statistics, Department of Community Medicine USM‐KLE International Medical School Belagavi India

**Keywords:** Dental plaque, dentition, epidemiology, gingivitis, oral hygiene, sociodemographic variables, survey

## Abstract

This cross‐sectional survey study evaluated oral hygiene habits in conjunction with whole mouth examinations for dental plaque and gingivitis among adults in India. Subjects across several age groups who provided informed consent [220 male and 158 female (mean age 30.9 years)] were enrolled. All enrolled subjects were interviewed for oral hygiene practices and evaluated by the Turesky modification of the Quigley‐Hein and the Löe‐Silness methods for dental plaque and gingivitis, respectively. Evaluations included oral hygiene parameters, prevalence of dental plaque and gingivitis, and regional differences within the dentition for dental plaque and gingivitis. Results from this study indicate that most subjects (97%) utilized a toothbrush and toothpaste for oral hygiene with a majority (92%) using their right hand to brush their teeth. While 29% reported two or more episodes of daily oral hygiene, a majority (53%) brushed their teeth once daily. Utilization of dental floss and mouthwashes were reported by approximately 1% of this population, and most (73%) reported no dental visits in the preceding 5 years. Whole mouth plaque and gingival scores (average ± standard deviation) for this population were 2.47 ± 0.55 and 1.19 ± 0.31, respectively, with no significant differences between either gender (P > 0.05). Significant correlations (r > 0.44) were observed between plaque and gingival scores for the entire sample, either gender or between age groups (P < 0.001). Analyses indicate that anterior teeth demonstrated lower average scores for dental plaque and gingivitis than posterior and molar regions (P < 0.05). Education was associated with higher plaque and gingival scores: plaque scores [odds ratios; 95% confidence interval; 1.23; 1.01–1.50 and gingival scores odds ratios 1.25; 1.02–1.54]. In summary, results from this study demonstrate the prevalence of dental plaque and gingivitis in the general population and their relationships with demographic characteristics. They reinforce examinations of posterior regions that consistently harbor more plaque and corresponding gingivitis in evaluations of oral health.

## Introduction

Oral health priorities seek to reduce the negative impacts of oral diseases and their influences on overall health (Dye 2012; Milgrom and Reisine 2000; Petersen 2009). Common oral diseases include caries and inflammatory conditions of the gingiva that affect oral health and may lead to tooth loss (Kornman 2008; Marsh 2012; Milgrom and Reisine 2000). A substantial literature has been instrumental in delineating the etiology and progression of these oral conditions (Petersen 2009; Scannapieco 1998; Socransky and Haffajee 2005). Epidemiological studies demonstrate the global nature of these conditions with a widespread prevalence (Dye 2012). Consequently, efforts to reduce the negative influences of these diseases on oral health represent important priorities for dental health‐care providers (Milgrom and Reisine 2000; Petersen 2009).

Dental plaque is a widely recognized factor in the initiation and progression of a variety of oral diseases (Berezow and Darveau 2011; Marsh 2012). Plaque, a natural biofilm, is commonly recovered from oral surfaces and comprises a diverse array of organisms (Socransky and Haffajee 2005). An unimpeded accumulation of dental plaque on the gingival margin triggers inflammatory effects that can become chronic (Kornman 2008; Rüdiger et al. 2002). Changes in protein profiles and microbial population shifts are reported during the clinical transition from health to inflammatory diseases such as gingivitis and periodontal disease (Rüdiger et al. 2002; Socransky and Haffajee 2005). Based on experimental and epidemiological investigations, dental professionals recommend effective oral hygiene to control the dental plaque and accumulated inflammatory components to maintain optimal oral health (Claydon 2008; Marsh 2012). Whereas the effects of dental plaque on the oral health of individuals are acknowledged, recent studies have assessed influences of poor oral health on overall health (de Oliveira et al. 2010). Taken together, these public health impacts represent important concerns for dental care providers (Scannapieco 1998; Schiavo, 2011).

The global epidemiology of common dental conditions is a reminder for effective dental programs (Dye 2012; Petersen 2009). While a vast literature explains the role of dental plaque in oral diseases (Claydon 2008; Marsh 2012; Milgrom and Reisine 2000; Socransky and Haffajee 2005), fewer studies describe the prevalence of dental plaque and gingivitis in populations. Recent investigations indicate average gingival scores among selected adult groups from different countries ranged from 0.99 to 1.23 (Li et al. 2010; Röthlisberger et al. 2007; Zhang et al. 2010). However, the published literature has few reports describing the prevalence of these common conditions among Indian adults or the distribution of plaque and gingivitis within the dentition. Whereas this information is important from a public health perspective, they are also relevant while evaluating therapeutic strategies to control these common oral conditions.

Accordingly, the present cross‐sectional survey study evaluated the general prevalence of dental plaque and gingivitis among adults (>18 years) in a population from India. Included in this study was an assessment of individual‐level factors, sociodemographic factors, oral health behavior, and dimensions of the home and family environments. Thus, the aims of this investigation were to (1) examine the prevalence of dental plaque and gingivitis among adult subjects; (2) determine distributions of plaque and gingivitis within the dentition; and (3) estimate common oral health practices to evaluate the contributions of these indications on dental plaque and gingivitis in this population.

## Materials and Methods

The present cross‐sectional study was conducted among adults (>18 years) after the study protocol was approved by the ethical review board of the SDM Dental College and Hospital, Dharwad, India. Prospective subjects from the local area provided written voluntary informed consent prior to enrollment. Three hundred seventy‐eight adults over the age of 18 years were recruited for this study.

Following enrollment, all subjects were interviewed for household demographics, social and economic characteristics, level of education, residential setting, and disabilities. In addition, an interview evaluated food habits, utilization of dental services, exposure to fluoridated water, oral hygiene habits (use of fluoride toothpaste and toothbrush, dental floss, mouth rinse use, and frequency of toothbrush replacement), frequency of routine and other dental visits, and smoking. Other variables recorded were gender, age in years, and region of residence. All data were collected by dental examiners by questionnaire with multiple choice questions.

## Clinical Evaluations

Clinical examinations for dental plaque and gingivitis were conducted by a calibrated examiner under constant lighting conditions. In initial tests and retest assessments among a group of eight subjects, the examiner demonstrated 99% reliability for both indices. Whole‐mouth evaluations for gingivitis and dental plaque were evaluated by the Löe–Silness (Loe 1963) and Turesky Modification of the Quigley–Hein (Turesky et al. 1970), respectively. The scoring scheme for the Löe–Silness gingivitis index is as follows:

0 = absence of inflammation
1 = mild inflammation – slight change in color and little change in texture
2 = moderate inflammation – moderate glazing, redness, edema and hypertrophy. Tendency to bleed upon probing
3 = severe inflammation – marked redness and hypertrophy. Tendency to spontaneous bleeding

The scoring scheme for the Turesky Modification of the Quigley–Hein dental plaque index is as follows:

0 = no plaque
1 = separate flecks of plaque at the cervical margin of the tooth
2 = a thin continuous band of plaque (up to 1 mm) at the cervical margin of the tooth
3 = a band of plaque wider than 1 mm but covering less than one‐third of the crown of the tooth
4 = plaque covering at least one‐third but less than two‐thirds of the crown of the tooth
5 = plaque covering two‐thirds or more of the crown of the tooth

Clinical examinations were conducted by the calibrated examiner, and a dental assistant recorded all results on appropriate forms.

## Statistical Analyses

All collected data were entered onto Excel spreadsheets and exported to SAS (Cary, N.C. USA) for statistical analysis. Descriptive statistics reported on collected data along with frequency distribution of demographic results. Frequencies for each evaluation were determined. Statistical analyses by *t*‐test and analysis of variance determined statistical differences. Statistical analyses were two sided with significance reported at *P* < 0.05.

## Results

A summary of subject demographics and oral hygiene habits from 378 adults [220 men and 158 women; average age 30 years] evaluated is shown in Table 1. A majority of subjects lived in urban or semi‐urban locations, utilized municipal water, and reported a high school education or more. Most used a toothpaste and a toothbrush (97%) for once daily oral hygiene (53%) and were right handed (92%). In this population, 60% used a half head of toothpaste for brushing with many reporting low utilization of mouth rinses and dental floss and 73% reporting no dental visits in the past 5 years.

**Table 1:** Sociodemographic variables of study population.

Characteristics	No. of respondents	% of respondents
Gender		
Male	220	58.20
Female	158	41.80
Age		
Mean	30.9	
SD	10.2	
Marital status		
Unmarried	155	41.01
Married	223	58.99
Residential setting		
Single (lives alone)	74	19.58
Married living with spouse	117	30.95
Family (lives as part of a large family)	186	49.21
Institution	1	0.26
Location		
Rural	34	8.99
Semi‐urban	158	41.80
Urban	186	49.21
Education		
Professional or honors	22	5.82
Graduate or post graduate	153	40.48
Intermediate or post high school diploma	75	19.84
High school certificate	82	21.69
Middle school certificate	19	5.03
Primary school certificate	17	4.50
Illiterate	10	2.65
Occupation		
Professional	67	17.72
Semi‐profession	24	6.35
Clerical, shop owner	25	6.61
Skilled worker	27	7.14
Semi‐skilled worker	43	11.38
Unskilled worker	49	12.96
Unemployed	143	37.83
Disabilities		
None	372	98.41
Physical	6	1.59
Drinking water		
Bottled water	5	1.32
Prepared at home	22	5.82
Tap water	348	92.06
Do not know	0	0.00
Some other sources	3	0.79
Household source drinking		
Town/municipal supply	263	69.31
Well water	112	29.63
Do not know	3	0.79
Oral Health Perceptions (a) (Experienced toothache/oro‐facial pain/food avoidance)		
Yes	170	44.97
No	208	55.03
Oral Health Perceptions (b) (Self‐perceived need for extraction or filling)		
Yes	194	51.32
No	184	48.68
Oral Health Perceptions (c) (Self‐rated oral health)		
Yes	343	90.74
No	35	9.26
Dentist visit		
Problem	342	90.48
Checkup	20	5.29
Other	16	4.23
Frequency of dental visit		
Every 6 months	18	4.76
Every 1–2 years	84	22.22
Every 5+ years or Never/problem only	276	73.02
Last visit to dentist		
Past 12 months	79	20.90
Between 1–2 years	62	16.40
Every 5+ years or never/problem only	209	55.29
Do not know	28	7.41
Avoid dental care		
Yes	120	31.75
No	258	68.25
How do you brush		
Tooth brush	367	97.09
No toothbrush	9	2.38
Natural	2	0.53
Frequency of tooth brushing habits		
Less than once per day	64	16.93
Once per day	203	53.70
Twice per day	108	28.57
More than twice a day	3	0.79
Handedness of brushing		
Right hand	349	92.33
Left hand	25	6.61
Both hands	4	1.06
Brush replacement		
A (<3 months)	228	60.32
B (3–6 months)	134	35.45
C (up to 1 year)	8	2.12
D (>1 year)	5	1.32
E (cannot say)	3	0.79
Dentifrice used (amount of paste used)		
A (full head)	127	33.60
B (half head)	229	60.58
C (unsure)	6	1.59
D (mixed use)	16	4.23
Dental floss		
Yes	4	1.06
No	374	98.94
Mouth rinse		
Yes	4	1.06
No	371	98.15
Occasionally	3	0.79
Grand total	378	100.00

SD, standard deviation.

The average whole‐mouth dental plaque and gingival scores of the entire population and among male and female subjects are shown in Table 2. These clinical evaluations were conducted by one dentist who demonstrated 99% reliability [data not shown]. Average scores for dental plaque and gingival index scores for the entire population were 2.47 and 1.19, respectively. Whole‐mouth plaque scores of male and female subjects were 2.48 and 2.46, respectively, while whole‐mouth gingival index scores for men and women were 1.20 and 1.19, respectively, with analyses demonstrating no significant differences between genders for each evaluated parameter (*P* > 0.05).

**Table 2:** Whole mouth clinical scores (mean ± SD).

Clinical index	Group	Number of subjects	Mean±SD
Dental plaque	All subjects	378	2.47±0.55
	Male	220	2.48±0.54‡
	Female	158	2.46±0.57‡
Gingival scores	All subjects	378	1.19±0.31
	Male	220	1.20±0.30‡
	Female	158	1.19±0.32‡

SD, standard deviation.

‡ No statistically significant differences between either gender (*P* > 0.05).

Table 3 provides whole‐mouth clinical scores within each age group. Average plaque scores ranged from 2.45 to 2.5, while gingival scores ranged from 1.17 to 1.27 with no significant differences for each parameter between evaluated age groups (*P* > 0.05). Significant correlations (*r* > 0.44) were observed between plaque and gingival scores for the entire sample, either gender or between age groups (*P* < 0.001) [data not shown].

**Table 3:** Whole‐mouth clinical scores within age groups (mean ± SD).

Age group	Dental plaque scores‡	Gingival index scores‡
18–27	2.49±0.55	1.17±0.30
28–37	2.45±0.57	1.19±0.31
38–47	2.46±0.54	1.24±0.31
48+	2.50±0.53	1.27±0.32

SD, standard deviation.

‡ No statistically significant differences between each age for either clinical evaluation (*P* > 0.05).

**Figure 1:**
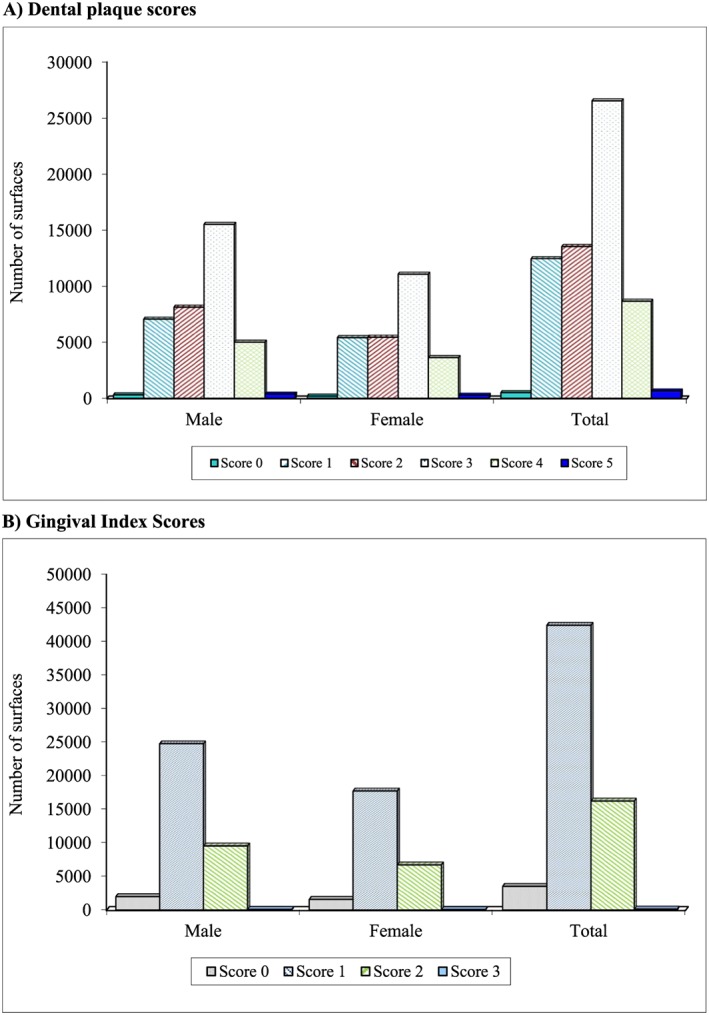
Distribution of individual clinical scores by gender and in the entire population. (A) Dental plaque scores; (B) gingival index scores.

The distribution of each dental plaque score in the entire population and by gender is shown in Figure 1A. A large number of surfaces registered a dental plaque score of 3 with more than 25,000 observations. Dental plaque scores of 1 and 2 were less frequent in this population, with even fewer sites harboring plaque scores of 4. In this population, sites with a plaque score of 5 were only found in 645 sites with 370 and 275 surfaces in men and women, respectively, with this score. Few surfaces were plaque free.

The distribution of gingival index scores in the population and by gender are shown in Figure 1B. Surfaces with scores of 1 were the most common with more than 42,000 surfaces observed in the entire population comprising more than 24,000 and 17,000 among men and women, respectively. Surfaces with gingival scores of 2 were less common than those that registered a score of 1. Clinical observations indicate that a gingival score of 3 was only found on 127 surfaces of the entire population on 77 and 50 surfaces of male and female subjects, respectively. No gingivitis was observed in 5.67% or 3525 surfaces representing 1961 and 1564 surfaces in men and women, respectively.

**Figure 2:**
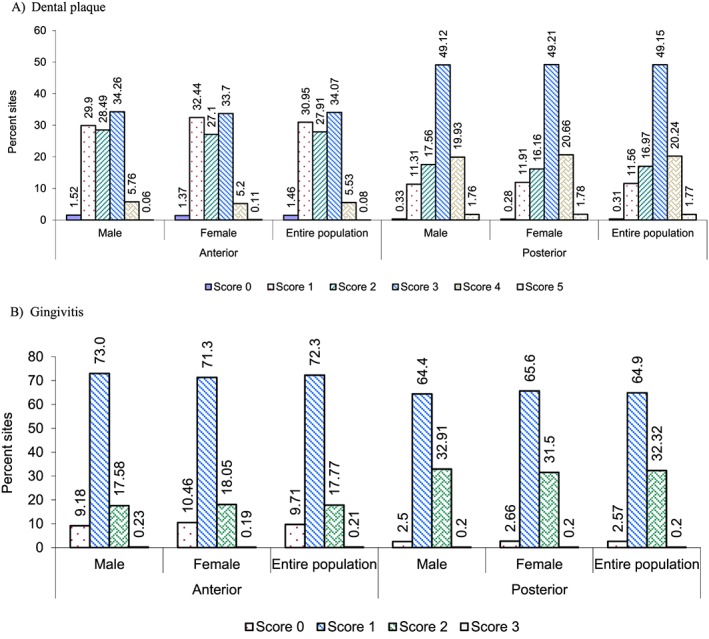
Frequency distribution of individual clinical scores on the anterior and posterior regions of the dentition. (A) Dental plaque; (B) gingivitis.

Frequency distributions of clinical scores (plaque and gingival index) on the anterior and posterior regions of the dentition in each gender and for both clinical parameters are presented in Figures 2A and 2B. Plaque scores of 1–3 were common on anterior teeth and were found on 27–34% of evaluated surfaces. Anterior surfaces with scores of 4 and 5 were less frequent and observed on approximately 5.5% and 0.08% of evaluated surfaces, respectively. Plaque scores on posterior surfaces demonstrated a different frequency distribution than anterior sites. A plaque score of 3 was most common on posterior sites and observed on 49% of evaluated sites. A score of 4 was found on 20% of posterior teeth and were more frequent than scores of 1 and 2 observed in 11–17% of surfaces. Less than 2% of posterior surfaces registered a plaque score of 5. Gingival index scores of anterior regions demonstrated *~*9% of sites with a score of 0, while a score of 1 was observed in 72% of sites and ~17% of sites demonstrating a score of 2. Gingival index frequencies of posterior regions indicate ~32% with a score of 2 and ~64% with a score of 1. Less than 3% of posterior sites recorded a gingival index score of 0.

Dental plaque and gingival index scores from the anterior and posterior regions for the entire population are shown in Table 4. Plaque and gingival scores were 2.12 and 1.09, respectively, for anterior regions, while scores on posterior regions were 2.83 and 1.30 for dental plaque and gingival scores, respectively. Scores for dental plaque were significantly lower on anterior teeth than posterior sites (*P* < 0.000). Correspondingly, plaque scores on anterior surfaces of male and female subjects were 2.13 and 2.09, respectively, and were significantly lower than corresponding scores on posterior regions, which were 2.82 and 2.84 for male and female subjects, respectively (*P* < 0.000). For the entire population, average gingival scores on anterior and posterior sites were 1.09 and 1.30, respectively, with significant differences between these sites (*P* < 0.000). For either gender, significantly lower gingival scores were observed on anterior surfaces (average score of 1.08–1.09) with posterior surfaces registering average scores of 1.31 and 1.29 among male and female subjects, respectively (*P* < 0.000). An additional analysis compared between gender the scores registered for either the anterior or the posterior regions (Table 4). Within each of these regions, there were no significant differences for dental plaque and gingivitis scores irrespective of gender (*P* > 0.05).

**Table 4:** Clinical scores in anterior and posterior regions of the mouth (average ± SD).

Clinical Index	Group	Number of subjects	Anterior		Posterior		*t*‐test (*P* value)
			Mean	SD	Means	SD	
Dental plaque	All subjects	378	2.12	0.64	2.83	0.57	0.0001*
	Male	220	2.13^a^	0.63	2.82^c^	0.55	0.0001*
	Female	158	2.09^a^	0.65	2.84^c^	0.58	0.0001*
Gingival scores	All subjects	378	1.09	0.36	1.30	0.30	0.0001*
	Male	220	1.09^b^	0.35	1.31^d^	0.30	0.0001*
	Female	158	1.08^b^	0.38	1.29^d^	0.30	0.0001*

SD, standard deviation.

* Statistically significant differences.

a‐d No significant differences between gender for evaluated clinical score (*P* > 0.05).

Analyses of anterior and posterior teeth within each age group for dental plaque and gingivitis are shown in Tables 5 and 6, respectively. Irrespective of age, analyses indicate significantly higher dental plaque and gingivitis scores on posterior regions than the corresponding anterior regions (*P* < 0.000).

**Table 5:** Plaque scores on anterior and posterior surfaces within age groups.

Age groups	Number of subjects	Anterior surfaces (Avg±SD)	Posterior surfaces (Avg±SD)	*t*‐test (*P* value)
18–27	163	2.16±0.65	2.82±0.55	0.0001*
28–37	117	2.09±0.64	2.81±0.58	0.0001*
38–47	66	2.07±0.63	2.85±0.55	0.0001*
48+	32	2.10±0.57	2.88±0.63	0.0001*
Entire population	378	2.12±0.64	2.83±0.57	0.0001*

Avg, average; SD, standard deviation.

* Statistically significant differences

**Table 6:** Gingival scores on anterior and posterior surfaces within age groups.

Age groups	Number of subjects	Anterior surfaces (Avg±SD)	Posterior surfaces (Avg±SD)	*t*‐test (*P* value)
18–27	163	1.07±0.36	1.26±0.28	0.0001*
28–37	117	1.08±0.36	1.30±0.31	0.0001*
38–47	66	1.11±0.37	1.37±0.30	0.0001*
48+	32	1.14±0.36	1.4±0.32	0.0001*
Entire population	378	1.09±0.36	1.30±0.30	0.0001*

Avg, average; SD, standard deviation.

* Statistically significant differences.

**Figure 3:**
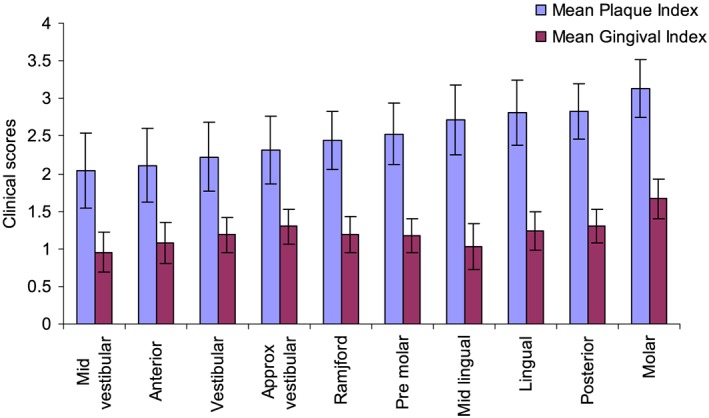
Dental plaque and gingival scores within distinct regions of the dentition (average ± standard deviation).

A summary of the average scores with distinct regions of the dentition is shown in Figure 3. Lower plaque and gingival scores were observed in mid‐vestibular and anterior sites, while lingual, posterior, and molar sites registered higher levels. Analyses indicate that anterior teeth demonstrated significantly lower average scores for dental plaque and gingivitis than posterior and molar regions (*P* < 0.05).

Using step‐wise logistic regression, education demonstrated a significant association with clinical outcomes for dental plaque and gingivitis. Lower education demonstrated a significant association with higher scores of dental plaque and gingival index (*P* < 0.05). Similarly, the level of parental education demonstrated a significant relationship with higher gingival scores in the posterior region (*P* < 0.05). Lower education levels, that is, those with less than middle school education, were associated with higher plaque and gingival index scores: plaque scores [odds ratios (OR); 95% confidence interval (95% CI); 1.23; 1.01–1.50 and gingival index scores OR 1.25; 1.02‐1.54] representing statistically significant relationships.

## Discussions

The present study was aimed at gathering data regarding oral health, whole‐mouth evaluations for dental plaque and gingivitis, among a sample of adult subjects in India. While studies on oral health are available from selected groups in India (Ameer et al. 2012; Bharateesh et al. 2012; Bhagyajyothi and Pushpanjali 2011; Chandra Shekar and Reddy 2011; Gupta et al. 2012; Gopinath 2010; Jain et al. 2009; Jain et al. 2012; Mahesh Kumar et al. 2005; Oswal 2013; Poudyal et al. 2010; Singh and Tuli 2013) to our knowledge, outcomes evaluated in this study remain unreported from the general population.

It is important to mention several aspects of this study that were standardized. Whole‐mouth clinical evaluations were conducted with the Turesky modification of Quigley–Hein and the Löe–Silness Index representing well‐recognized approaches to evaluate oral hygiene (Dowsett et al. 2002; Williams et al. 2004; Zhang et al. 2010). Advantages of these indices include their wide application as the “gold standard” for a comprehensive assessment of the entire mouth as presented in many previous studies (Goyal et al. 2005; Poyato‐Ferrera et al. 2003; Williams et al. 2004) in contrast with partial mouth evaluations (Dowsett et al. 2002; Ericsson et al. 2012; Holtfreter et al. 2009; Owens et al. 2003). Clinical evaluations were conducted by a calibrated clinical examiner who demonstrated 99% reliability in the clinical indices.

Subjects evaluated in this study were residents of the local area and drawn from the general population. They were not selected from individuals seeking professional care (Al‐Otaibi et al. 2003b) or individuals belonging to selected groups or subjects of one gender (Bhagyajyothi and Pushpanjali 2011; Jain et al. 2009; Needleman et al., 2013). Unlike other studies, there were no preparatory or washout phases prior to oral examination (Furuichi et al. 1992; Sreenivasan et al. 2010) or any oral hygiene instructions prior to the examination. Subjects did not alter their diet or routine habits to reduce the influences of these parameters on dental plaque (Signoretto et al. 2006). Consistent with other studies, a majority of subjects reported brushing their teeth once daily (Gopinath 2010; Oswal 2013; Singh and Tuli 2013) and during interviews prior to oral examination indicated no prior participation in clinical studies or other investigations to further reduce the influences of these variables on evaluated parameters. While the population was homogeneous for ethnicity, differences in socioeconomic status and habitat were noted. Demographic features of study subjects indicated variations in cultural and dietary practices. A large number of subjects reported no recent dental visits and utilized dental services only in the case of pain or other emergencies, corroborating previous observations (Kumar et al. 2005; Poudyal et al. 2010) representing low utilization of dental services. While several factors such as proximity to dental clinics and affordability remain significant factors, it is also important to highlight the need for dental education. Together, these demographic observations are significant, because the observed results for dental plaque and gingivitis reveal their natural distribution within the mouth with few influencing parameters.

Salient outcomes from this study demonstrate that average whole‐mouth plaque and gingival scores for this population were 2.47 and 1.19, respectively. The average results for gingivitis had similarity to those reported in previous studies of 0.99 from Saudi Arabia (Al‐Otaibi et al. 2003a), 1.23 from Swiss recruits (Röthlisberger et al. 2007), 1.2 from The Gambia (Jordan et al. 2011), 1.05 from USA (Li et al. 2010), and 1.1 from China (Zhang et al. 2010) representing populations from different regions. Included in the present investigation were frequencies of plaque and gingival index scores representing parameters generally not reported. Whole‐mouth scores for dental plaque and gingivitis were ~2.4 and ~1.2, respectively, with no remarkable differences between the age groups or gender. These observations contrast other reports that indicate lower gingivitis scores in female subjects (Idrees et al. 2014) but have similarity with reports suggesting a relationship and education (Ababneh et al. 2012). A majority of sites registered plaque scores between 1 and 3 with a score of 3 being the most common irrespective of gender in the entire mouth. Sites with scores of 4 and 5 were less frequent with few sites free of plaque for the entire population. Gingivitis is prevalent widely in many populations and has been widely reported among adults (Angst et al. 2013; Rebelo et al. 2009), including special populations such as elite athletes (Needleman et al. 2013). Commonly observed gingivitis scores in the present study were 1 and 2. Sites without gingivitis were also observed. Similar to some previous reports, this study demonstrated no differences in gingivitis between gender (Jordan et al. 2011) but were different from several other studies that demonstrate lower scores among female subjects (Furuta et al. 2011; Mizutani et al. 2012). While reasons for these observations remain unclear, it is possible that differences in dental behaviors and attitudes represent likely reasons for these observations. In addition, many in the evaluated population reported atleast a middle school education and comprised community‐dwelling adults who were not seeking dental care during the study period. Analyses indicate that whole‐mouth gingival scores were 1.19 for women and 1.20 for men with no differences noted between genders. Gingival scores within age groups showed minor differences and were between 1.17 and 1.27 similar to previous observations (Lang et al. 2009) and different from others reporting increasing scores with age (Ababneh et al. 2012).

For the entire population, the sites that scored the least for both plaque and gingival indices were the mid‐vestibular regions. Progressive increases in these scores were observed in different areas of the mouth with anterior sites demonstrating one of the least scores. Consistently higher scores were observed in the posterior regions with molar teeth yielding the highest scores for either index. Additional analyses of anterior or posterior regions indicate no significant differences between either gender for either dental plaque or gingivitis. Dental plaque levels from anterior regions were 2.1 and contrasted with 2.8 observed at posterior sites. Similarly, gingivitis scores of anterior sites were 1.09 with posterior sites registering 1.30. Additional analyses indicate no age‐based differences for the indices recorded within the anterior or posterior sites.

Evaluations of the anterior and posterior regions for either gender indicate specific differences between these regions for dental plaque and gingivitis. Approximately 49% of the posterior surfaces demonstrated a plaque score of 3, while a score of 4 was reported in approximately 20% of the sites representing the most common observation. Scores of 1 and 2 were found on 11–17% of the sites and less than 2% of the sites registered a score of 5. Few sites were entirely plaque free in either the anterior or posterior regions representing differences from previous studies (Lang et al. 2009). Analysis of the anterior sites demonstrated a different pattern for dental plaque scores. Scores of 1–3 were most common and observed on 27–34% of sites with less than 6% of surfaces registering a score of 4. Less than 0.1% of the surfaces registered a score of 5 with these observations contrasting results from posterior surfaces. Differences between the regions have been reported previously in studies that included other procedural steps (Angst et al. 2013; Farina et al. 2013; Furuichi et al. 1992; Prasad et al. 2011; Ramberg et al. 1994; Ramberg et al. 1995; Sreenivasan et al. 2010). Results from this investigation also demonstrate differences in gingivitis scores within the mouth. No gingivitis was observed at 9% of anterior sites in comparison with 2.57% of posterior sites. A gingivitis score of 1 was observed at 72% of anterior sites, while 64% of posterior sites registered this score. Sites with a gingivitis score of 2 were observed at a higher frequency in posterior regions and observed in 32% of sites. Recent research reports that sites with persistent and long‐standing gingivitis progress to periodontitis along with sites with gingivitis scores of 2 or more as a clinically relevant risk factor for tooth loss (Lang et al. 2009). In this study, sites with a gingivitis core of 2 were more frequent than those reported previously from a Canadian survey (Health Canada 2010) and other areas (Australian Research Centre for Population Oral Health, The University of Adelaide, South Australia 2009). Furthermore, recent longitudinal research by Soder et al. 2015 indicates an association between years of gingival inflammation and a risk of stroke. Taken together, these observations report a latent or unreported inflammatory burden and a comprehensive survey of community‐dwelling adults from a mid‐size city. These results are relevant from a practical standpoint for prevention of future conditions. At the conclusion of the investigation, all subjects were provided an instructional program on oral health developed by investigators from the dental college. It may be useful to follow up these subjects in a future investigation to evaluate the effect of instructional programs on oral health.

## Conclusion

Results from the present study are in congruence with those in previous reports including those that evaluated children (Krisdapong et al. 2012), other populations (Jones et al. 2011), including special populations (Needleman et al. 2013). Furthermore, these observations are noteworthy from the standpoint of health policies and oral health evaluations. Results reaffirm regional differences within the mouth for dental plaque and gingivitis reported previously (Angst et al. 2013; Claydon 2008; Cumming and Löe 1973; Furuichi et al. 1992; Nguyen et al. 2010; Prasad et al. 2011; Ramberg et al. 1994; Ramberg et al. 1995; Sreenivasan et al. 2010). These observations are significant from the stand point of preventative programs and highlight the need for whole‐mouth examinations. In addition, these differences highlight a need for effective oral hygiene in the posterior regions that register higher amounts of dental plaque and gingivitis irrespective of gender.

## Conflict of Interest

P.K. Sreenivasan is an employee of Colgate‐Palmolive Company.
